# Knowledge, Attitudes and Practises Amongst Nursing Staff in a Tertiary Hospital Regarding Subcutaneous Anticoagulant Administration in Jiaxing City, Southeast China

**DOI:** 10.1002/nop2.70687

**Published:** 2026-07-14

**Authors:** Qianjuan Wang, Jiali Lu, Zhihong Zhu, Yuwei Wang, Liangfeng Zhu, Lan Shen

**Affiliations:** ^1^ Department of Hepatobiliary Surgery Jiaxing University Affiliated Hospital (Jiaxing First Hospital) Jiaxing China; ^2^ Department of Vascular Surgery Jiaxing University Affiliated Hospital (Jiaxing First Hospital) Jiaxing China; ^3^ Department of Nursing Jiaxing University Affiliated Hospital (Jiaxing First Hospital) Jiaxing China; ^4^ College of Medicine, Jiaxing University Jiaxing China; ^5^ School of Nursing Changsha Medical University Changsha China; ^6^ Department of Quality Management Jiaxing University Affiliated Hospital (Jiaxing First Hospital) Jiaxing China

**Keywords:** anticoagulants, attitude, injection, knowledge, nurses, practise

## Abstract

**Aim:**

This study evaluates the knowledge, attitude and practise (KAP) of nursing staff in Jiaxing City, Southeast China, regarding the subcutaneous administration of anticoagulants, a critical aspect of managing venous thromboembolism (VTE).

**Design:**

A cross‐sectional study.

**Review Methods:**

The study was conducted at a tertiary hospital in Jiaxing, using a self‐administered questionnaire to collect demographic data and assess KAP related to subcutaneous anticoagulant administration amongst nursing staff.

**Results:**

The study included 545 nursing staff with a mean age of 32.11 years. The mean (SD) scores for knowledge, attitude and practise were 56.23 (6.21), 47.35 (4.35) and 67.03 (6.15), respectively, with possible score ranges of 0–72, 10–50 and 14–70. In the knowledge section, the highest accuracy was observed for the item ‘Complications that may occur during subcutaneous anticoagulant injection include: Subcutaneous bleeding’, with an accuracy rate of 99%. However, only 16.88% of participants correctly identified the item ‘Absolute contraindications for subcutaneous anticoagulant injection include: Impaired liver or kidney function’ as incorrect. Spearman correlation and path analyses indicated positive associations amongst KAP dimensions based on self‐reported data. Nursing staff generally demonstrated relatively adequate knowledge, positive attitudes and self‐reported proactive practises regarding subcutaneous anticoagulant administration, although certain knowledge deficiencies were observed and may benefit from targeted educational interventions.

**Implications for the Profession:**

The findings highlight the need for ongoing education and training programmes to enhance the knowledge of nursing staff, particularly related to contraindications in anticoagulant therapy. These self‐reported results suggest that strengthening standardised nursing education may contribute to improved patient safety and care quality in clinical practise, although outcomes were not directly measured.

## 1 Introduction

Venous thromboembolism (VTE) is characterised by the formation of blood clots that obstruct normal blood flow. This condition includes both deep vein thrombosis (DVT) and pulmonary embolism (PE), where a clot forms in a deep vein and may dislodge, travelling to the lungs (Speth 2023). VTE can affect individuals in various healthcare settings, including inpatient and outpatient populations with chronic heart failure (CHF), cancer and obesity. Despite its importance, VTE is often unrecognised, leading to severe long‐term complications such as post‐thrombotic syndrome (PTS) following DVT and post‐PE syndrome and persistent thromboembolic pulmonary hypertension after PE, all of which contribute to increased mortality rates (Klok et al. 2014; Phillippe 2017; Martin et al. 2021; Rosovsky et al. 2023). The exact incidence of VTE remains unclear. Estimates suggest that in North America, the annual incidence rate of VTE in the general population is approximately 0.1%, with PE accounting for roughly one‐third of these cases (Bartholomew 2017; Guntupalli et al. 2023).

Anticoagulant therapy plays a central role in managing conditions such as DVT and PE, particularly in patients with comorbidities like CHF, cancer and obesity (Kruger et al. 2019a, 2019b). The selection of specific anticoagulants and the determination of treatment duration are complex, influenced by factors such as the clinical presentation, underlying causes of the VTE event, the patient's bleeding risk and their preferences (Tritschler et al. 2018). Furthermore, it is important to recognise that anticoagulant therapy increases the risk of spontaneous bleeding and bleeding during surgical procedures or interventions. Patients may also experience complications from anticoagulant injections, such as bruising, hematoma formation and pain at the injection site, which can have emotional effects on patients, their families and healthcare providers (Koscielny et al. 2020; Mohammady et al. 2017; Hadley et al. 1996; Wooldridge and Jackson 1988). In this context, healthcare providers—particularly nursing staff—play a critical role in managing and preventing VTE through effective anticoagulant administration, ensuring patient compliance and improving treatment outcomes (Mohammady et al. 2017; Robb and Kanji 2002; Zaybak and Khorshid 2008).

The Knowledge, Attitude and Practise (KAP) framework has been widely used to guide the planning, implementation and evaluation of public health initiatives (Fan et al. 2018; Muleme et al. 2017). Knowledge refers to the awareness and understanding of specific information, indicating a direct comprehension of concepts (Liao et al. 2022). Attitude refers to the positive or negative evaluation of an object (Ajzen and Fishbein 2000). Practise involves habitual actions shaped by prevailing social norms and beliefs (Bourdieu 1990). The efficacy and safety of subcutaneous anticoagulant administration depend not only on the pharmacological properties of the drugs but also on the KAP of healthcare professionals, especially nurses (Malik et al. 2015).

In China, challenges such as improper anticoagulant prescriptions and the lack of long‐term sustainability and patient compliance with anticoagulant treatments have been identified. These issues result from a complex interaction amongst physicians, patients and the anticoagulants themselves (Shang et al. 2022). Previous studies have shown variability in practises amongst nursing staff regarding anticoagulant administration (Robb and Kanji 2002; Palese et al. 2013; Yılmaz et al. 2020; Pourghaznein et al. 2014), and these inconsistencies may negatively influence the safety and effectiveness of anticoagulant care (Li et al. 2021). However, existing evidence remains limited in systematically exploring how knowledge, attitudes and self‐reported practises are interrelated within the context of subcutaneous anticoagulant administration, particularly amongst nursing staff in tertiary hospitals in Southeast China. Therefore, this study aims to evaluate the KAP of nursing staff in tertiary hospitals regarding subcutaneous anticoagulant administration and to further explore the associations amongst these domains using a structural framework.

## 2 Methods

The study was carried out after the protocol was approved by the hospital's Medical Ethics Committee (2023‐KY‐655). All methods were performed in accordance with the relevant guidelines. All procedures were performed in accordance with the ethical standards laid down in the 1964 Declaration of Helsinki and its later amendments, and informed consent was obtained from all participants.

### 2.1 Study Design and Participants

This cross‐sectional study was conducted from November to December 2023 in Jiaxing City, Southeast China. The study participants were nurses from a tertiary hospital. Inclusion Criteria: (1) Nurses on duty; (2) possessing a professional nursing licence. Exclusion Criteria: (1) Nurses without experience in subcutaneous anticoagulant administration; (2) nurses who submitted invalid questionnaires.

### 2.2 Questionnaire and Data Collection

The questionnaire was designed based on previous studies (Haut et al. 2018; Lenchus 2011; Murphy et al. 2018; Schmerge et al. 2018) and the ‘*Expert Consensus on Nursing Standards for Subcutaneous Injection of Anticoagulant Agents*’ by the China Intravenous Intervention Alliance, Professional Committee on Peripheral Vascular Intervention, Chinese College of Interventionalists. After the initial design, modifications were made based on feedback from four senior experts (three experts in haematology nursing and one expert in nursing management) and a pilot study involving 39 participants was conducted. The Cronbach's α coefficient for the pilot study was 0.8831, indicating acceptable internal consistency. However, other psychometric properties of the instrument, such as construct validity and test–retest reliability, were not formally assessed in this study, which may limit comprehensive evaluation of the questionnaire's measurement performance.

The final questionnaire, in Chinese, consists of four dimensions: demographic characteristics, knowledge dimension, attitude dimension and practise dimension. The knowledge dimension includes 12 questions, with a total of 36 items. The first 11 questions are scored 2 points for a correct response and 0 points for an incorrect or unclear response. The 12th question is scored as 2 points for full understanding, 1 point for partial understanding and 0 points for lack of understanding. The total score ranges from 0 to 72 points.

The attitude dimension includes 10 questions and uses a five‐point Likert scale, ranging from ‘strongly agree’ (5 points) to ‘strongly disagree’ (1 point). The total score ranges from 10 to 50 points. The practise dimension includes 14 questions, also using a five‐point Likert scale from ‘always’ (5 points) to ‘never’ (1 point), with a total score ranging from 14 to 70 points. Participants' overall KAP scores were categorised based on a modified Bloom's cutoff criteria, whereby scores below 60% were classified as ‘poor’, scores between 60% and 80% as ‘moderate’, and scores exceeding 80% as ‘good’ (Bloom 1968). The full questionnaire used in this study is provided as a Supporting Information.

The electronic questionnaire was created on the ‘Wenjuanxing’ platform (https://www.wjx.cn/). A convenience sampling method was adopted, and the participants were recruited from one tertiary hospital in Jiaxing City, Southeast China. A dedicated research team was established for this study, and trained research staff were specifically responsible for questionnaire distribution and data management. After obtaining approval from the nursing department head, the survey link and corresponding QR code were distributed to nursing teams via the hospital's quality management department. Participants were informed of the study's objectives, anonymity and data confidentiality measures. Nurses could access the questionnaire by clicking the link provided in the group chat. To ensure data quality and completeness, each IP address was allowed to submit the questionnaire only once and all items were set as mandatory. If participants encountered difficulties whilst completing the questionnaire, members of the research team provided clarification and technical assistance without interfering with their responses. Upon completion of data collection, quality control procedures were implemented. Questionnaires were deemed invalid if they were incomplete, submitted in an unreasonably short time (less than 120 s), contained implausible responses (e.g., age of 150 years), or exhibited uniform response patterns (e.g., selecting the same option for all items).

### 2.3 Statistical Analysis

Statistical analysis was performed using STATA 14.0 (Stata Corp LP, USA) and AMOS 29.0 (IBM Corp., Armonk, New York, USA). Quantitative variables are presented as mean (SD), and categorical variables are presented as *n* (%). As the KAP scores were nonnormally distributed, intergroup comparisons were conducted using the Mann–Whitney U test for two‐group comparisons and the Kruskal–Wallis test for comparisons amongst three or more groups. Spearman correlation analysis was used to examine correlations amongst KAP scores. Path analysis was performed using a recursive structural equation model to examine the relationships amongst knowledge, attitude and practise. Based on the KAP theoretical framework, the model specified unidirectional paths from knowledge to attitude, from attitude to practise and a direct path from knowledge to practise. In addition, training experience and frequency of subcutaneous anticoagulant injection were included as exogenous variables. The model was estimated using maximum likelihood estimation, and direct, indirect and total effects were calculated. Model fit was evaluated using the root mean square error of approximation (RMSEA), standardised root mean square residual (SRMR), Tucker–Lewis index (TLI) and comparative fit index (CFI). In interpreting model fit indices, RMSEA values close to the conventional threshold were considered indicative of marginal but acceptable fit in the context of behavioural data and model complexity. A two‐sided *p*‐value of < 0.05 was considered statistically significant.

## 3 Results

### 3.1 Characteristics of Participants

A total of 632 individuals participated in this study. Amongst these, 63 cases had excessively short response times, 15 cases exhibited abnormal responses, 14 cases had incomplete questionnaire responses and 2 cases showed clear response patterns. As a result, 545 valid questionnaires were included in the final analysis. Specifically, a completion time of less than 120 s was considered excessively short. Abnormal responses referred to logically implausible data, such as reporting an age of 150 years. The mean age of participants was 32.11 years; the majority were female (95.60%). Of the participants, 87.52% had a Bachelor's degree or higher and more than half held junior titles. The majority (92.84%) reported having participated in training on ‘Subcutaneous Anticoagulant Administration’, and 96.33% confirmed that their hospital had internal guidelines for ‘Subcutaneous Anticoagulant Administration’. Nearly 45% of participants administered subcutaneous anticoagulant injections less than once a week (Table 1).

**TABLE 1:** Demographic characteristics and KAP.

*N* = 545	*N* (%)	Knowledge score		Attitude score		Practise score	
		Mean (SD)	*p*	Mean (SD)	*p*	Mean (SD)	*p*
Total score		56.23 (6.21)		47.35 (4.35)		67.03 (6.15)	
Age (years)	32.11 (6.56)						
Gender			0.286		0.093		0.269
Male	24 (4.40)	57.91 (4.95)		48.41 (3.39)		66.16 (5.66)	
Female	521 (95.60)	56.15 (6.25)		47.29 (4.38)		67.07 (6.17)	
Education			0.829		0.160		0.265
Associate degree and below	68 (12.48)	55.77 (7.66)		46.66 (4.79)		66.36 (6.14)	
Bachelor's degree and above	477 (87.52)	56.29 (5.97)		47.44 (4.27)		67.12 (6.14)	
Working experience (years)	10.17 (6.84)						
Department			0.010		0.004		0.024
Relevant department^a^	285 (52.29)	56.75 (6.10)		47.66 (4.28)		67.59 (5.14)	
Other	260 (47.71)	55.66 (6.27)		47 (4.39)		66.41 (7.04)	
Professional title			0.074		0.573		0.516
No title	44 (8.07)	55.52 (5.56)		46.13 (5.35)		65.77 (6.94)	
Junior	287 (52.66)	56.64 (6.42)		47.19 (4.41)		66.95 (6.47)	
Intermediate	193 (35.41)	55.82 (6.17)		47.69 (4.13)		67.24 (5.71)	
Senior	21 (3.85)	55.76 (4.50)		48.71 (1.38)		68.71 (2.19)	
Participated in training on ‘Subcutaneous Anticoagulant Administration’			< 0.001		< 0.001		< 0.001
Yes	506 (92.84)	56.60 (5.63)		47.65 (4.00)		67.53 (5.14)	
No	39 (7.16)	51.38 (10.2)		43.35 (6.31)		60.48 (11.90)	
Does your hospital have internal guidelines for ‘Subcutaneous Anticoagulant Administration’?			0.001		< 0.001		< 0.001
Yes	525 (96.33)	56.42 (5.99)		47.51 (4.12)		67.34 (5.43)	
No/not sure	20 (3.67)	51.20 (9.25)		42.90 (6.99)		58.90 (13.90)	
In the past year, how often have you performed subcutaneous injections of anticoagulants?			0.052		< 0.001		0.002
Less than once a week	243 (44.59)	55.56 (6.50)		46.69 (4.67)		65.98 (7.40)	
One to Four times a week	188 (34.50)	56.61 (6.38)		47.79 (4.01)		67.71 (4.91)	
Five times or more per week	114 (20.92)	57.02 (5.06)		47.99 (3.97)		68.13 (4.48)	

*Note:* Data are presented as *n* (%) or mean (SD). *p* values were calculated using the Mann–Whitney U test or Kruskal–Wallis test, as appropriate.

^a^ Including departments of Obstetrics and Gynaecology/Surgery/Orthopaedics/Oncology/ICU.

### 3.2 Knowledge, Attitude and Practise

The mean (SD) scores for knowledge, attitude and practise were 56.23 (6.21), 47.35 (4.35) and 67.03 (6.15), respectively, with possible score ranges of 0–72, 10–50 and 14–70, indicating favourable responses in all three areas (Table 1). The majority of respondents demonstrated adequate knowledge (55.6%), positive attitude (82.9%) and proactive practises (89.9%) regarding the subject (Table S1).

Significantly higher scores were observed in the knowledge (*p* = 0.010), attitude (*p* = 0.004) and practise (*p* = 0.024) dimensions amongst participants working in departments such as Obstetrics and Gynaecology, Surgery, Orthopaedics, Oncology, or ICU, compared to those in other departments. Participants who had received training on ‘Subcutaneous Anticoagulant Administration’ scored higher in the knowledge (*p* < 0.001), attitude (*p* < 0.001) and practise (*p* < 0.001) dimensions. Nurses working in hospitals with internal guidelines for ‘Subcutaneous Anticoagulant Administration’ also achieved higher scores in knowledge (*p* = 0.001), attitude (*p* < 0.001) and practise (*p* < 0.001). The more frequently nurses performed subcutaneous anticoagulant injections, the higher their scores in attitude (*p* < 0.001) and practise (*p* = 0.002) dimensions (Table 1).

In the knowledge dimension, the highest accuracy was found for the item ‘Complications that may occur during subcutaneous anticoagulant injection include: Subcutaneous bleeding’, with an accuracy rate of 99.45%. The lowest accuracy was observed for the item ‘Absolute contraindications for subcutaneous anticoagulant injection include: Impaired liver or kidney function’, with an accuracy rate of only 16.88% (Table S2). In the attitude dimension, more than 95% of participants strongly agreed or agreed that ‘Subcutaneous anticoagulant injection is effective in preventing or treating blood clots’ and ‘Subcutaneous anticoagulant injection technique is an important component of nursing skills’, and expressed interest in further studying subcutaneous anticoagulant injection (Table S3). In the practise dimension, over 70% of participants explained the indications and contraindications of subcutaneous anticoagulant injection to patients, informed patients (or their families) about potential risks and precautions to reduce anxiety, double‐checked patient identity, medication dosage and medication name, and assessed patients' physical condition (indications and contraindications), local conditions, psychological status and cooperation level (Table S4).

### 3.3 Correlations Amongst KAP

Correlation analysis showed positive associations between knowledge and attitude (*r* = 0.34, *p* < 0.001), knowledge and practise (*r* = 0.31, *p* < 0.001) and attitude and practise (*r* = 0.57, *p* < 0.001), as estimated using Spearman's rank correlation analysis (Table 2). Path analysis results showed that training experience was associated with knowledge (*β* = 4.98, *p* = 0.020) within the structural equation model based on self‐reported data, without implying causality. Amongst the KAP dimensions, knowledge was positively associated with both attitude (*β* = 0.29, *p* = 0.011) and practise (*β* = 0.13, *p* = 0.006) and attitude was positively associated with practise (*β* = 0.81, *p* = 0.011). These results suggest a potential indirect association between knowledge and practise based on self‐reported measures (Table 3), without implying causal effects. To assess the model fit, the RMSEA, SRMR, TLI and CFI were summarised in the Table S5, supporting the overall acceptability of the model. It should be noted that the RMSEA value indicated a borderline or marginal fit, which is not uncommon in complex structural equation models based on behavioural self‐reported data.

**TABLE 2:** Correlation analysis.

	Knowledge	Attitude	Practise
Knowledge	1.00		
Attitude	0.34 (0.26–0.41) (*p* < 0.001)	1.00	
Practise	0.31 (0.23–0.39) (*p* < 0.001)	0.57 (0.51–0.62) (*p* < 0.001)	1.00

*Note:* Correlation coefficients were estimated using Spearman's rank correlation analysis.

**TABLE 3:** Path analysis of factors associated with KAP scores.

Model paths	Total effects (95% CI)	*p*	Direct effects (95% CI)	*p*	Indirect effects (95% CI)	*p*
Knowledge → Attitude	0.29 (0.21–0.36)	0.011	0.29 (0.21–0.36)	0.011		
Knowledge → Practise	0.37 (0.24–0.47)	0.011	0.13 (0.04–0.23)	0.006	0.24 (0.15–0.31)	0.012
Attitude → Practise	0.81 (0.63–0.97)	0.011	0.81 (0.63–0.97)	0.011		
Participation in training → Knowledge	4.98 (1.39–8.20)	0.020	4.98 (1.39–8.20)	0.020		
Frequency of anticoagulant subcutaneous injection → Knowledge	0.50 (−0.16–1.20)	0.112	0.50 (−0.16–1.20)	0.112		
Participation in training → Attitude	1.46 (0.43–2.68)	0.018			1.46 (0.43–2.68)	0.018
Frequency of anticoagulant subcutaneous injection → Attitude	0.15 (−0.03–0.36)	0.077			0.15 (−0.03–0.36)	0.077
Participation in training → Practise	1.82 (0.48–3.44)	0.019			1.82 (0.48–3.44)	0.019
Frequency of anticoagulant subcutaneous injection → practise	0.18 (−0.03–0.48)	0.069			0.18 (−0.03–0.48)	0.069

*Note: p* values were obtained from path analysis based on a structural equation modelling framework. Therefore, all reported effects (direct, indirect and total) are model‐derived estimates rather than results from independent regression models. Total, direct and indirect effects are presented with 95% confidence intervals.

## 4 Discussion

This study demonstrated that nursing staff exhibited good KAP regarding subcutaneous anticoagulant administration. Specifically, the scores in the knowledge, attitude and practise sections were 56.23 (range: 0–72), 47.35 (range: 10–50) and 67.03 (range: 14–70), respectively. These findings are valuable for guiding the development of tailored educational interventions and practise guidelines, thereby improving the safety and efficacy of anticoagulant management.

In contrast, previous studies mainly focused on the prevention and alleviation of complications such as pain and bruising associated with subcutaneous anticoagulant administration (Yılmaz et al. 2020; Karadağ et al. 2023). Our study, however, focused on the assessment of nurses' KAP. Nearly all items received good responses, particularly in the attitude and practise dimensions. However, the knowledge domain raised some concerns. For example, although over 80% of participants were aware that the mechanisms of heparin's antithrombotic action include enhancing anticoagulant enzyme activity, inhibiting platelet function and enhancing protein C activity, nearly 67% mistakenly believed that heparin could promote vasodilation. Heparin mainly exerts its effect by inhibiting thrombin (FIIa) and/or FXa, potentiating heparin cofactor II, interacting with C1‐esterase inhibitor and interacting with tissue factor pathway inhibitor. Promotion of vasodilation, however, is not one of the mechanisms of heparin's anticoagulant action (Onishi et al. 2016). Additionally, only 16.88% of participants correctly identified that impaired liver or kidney function is not an absolute contraindication for subcutaneous anticoagulant injection. The mechanisms of heparin and the appropriate conditions for subcutaneous anticoagulant injection should be thoroughly understood by nursing staff through targeted training.

Approximately 75% of nursing staff in this study believed that ‘the longer the injection needle for anticoagulants, the greater the risk when injecting into the muscle layer. Therefore, shorter needles should be preferred whenever possible’. However, this perspective was incorrect. Although one study showed that the use of smaller needle sizes (28‐gauge, 1/2‐in. vs. 25‐gauge, 5/8‐in.) for unfractionated heparin (UFH) administration reduced the frequency and severity of injection‐related discomfort (Coley et al. 1987), a clinical trial by Robb et al. suggested that using a 30‐gauge, 5/16‐in. insulin syringe instead of a 26‐gauge, 3/8‐in. tuberculin syringe did not significantly reduce either hematoma size or pain (Robb and Kanji 2002). In clinical practise, needle size should be selected based on multiple factors (Yılmaz et al. 2020).

In addition, only about half of the study participants recognised that, besides VTE prevention and treatment, indications for subcutaneous anticoagulant injection include conditions such as diabetic nephropathy (Han et al. 2023). Hirudin has been used to improve diabetic nephropathy by inhibiting Gsdmd‐mediated pyroptosis (Han et al. 2023). Awareness of the indications for anticoagulant injection needs to be improved amongst nurses. Notably, 47.71% of participants worked in departments unrelated to the administration of subcutaneous anticoagulants. This may have negatively affected the KAP scores of the overall study sample. In hospitals with limited resources, the knowledge and skills of nurses in nonspecialised departments must be enhanced to properly administer subcutaneous anticoagulants. Furthermore, educational level and professional title may also influence KAP outcomes, although no significant differences were observed between participants with different educational backgrounds and professional titles in this study.

This study has several limitations. Path analysis indicated positive associations amongst KAP dimensions based on self‐reported data; however, causal inferences cannot be established due to the cross‐sectional design and measurement limitations. These findings suggest the importance of translating favourable KAP into clinical practise, particularly in subcutaneous anticoagulant administration, whilst acknowledging that practise outcomes were self‐reported rather than objectively verified. In addition, although internal consistency was acceptable in the pilot testing, the absence of comprehensive psychometric validation (e.g., construct validity, criterion validity and test–retest reliability) should be considered when interpreting the findings. Furthermore, the practise domain was entirely based on self‐reported responses, which may introduce reporting bias and limit the objectivity of behavioural assessment. From a nursing practise perspective, these findings emphasise the importance of strengthening standardised training for subcutaneous anticoagulant administration, particularly in reinforcing knowledge of contraindications, indications and safe injection techniques to reduce variability in clinical practise. In addition, the results support the value of integrating structured KAP‐based educational strategies into continuous nursing education programmes to improve medication safety and consistency of care. From a theoretical standpoint, the study further supports the applicability of the KAP framework in explaining variations in nursing‐related clinical behaviours, whilst also highlighting the limitations of self‐reported behavioural measures when interpreting observed associations.

## 5 Conclusion

In conclusion, nursing staff in this study showed relatively adequate knowledge, positive attitudes and self‐reported proactive practises regarding subcutaneous anticoagulant administration, whilst specific knowledge gaps were identified, such as the misconception about heparin‐induced vasodilation. These findings highlight the need for ongoing, focused education to address specific misunderstandings. This study contributes to understanding the KAP of nursing staff in clinical practise in the eastern regions of China. However, knowledge related to the mechanisms and indications of anticoagulants requires enhancement through targeted education and training.

### 5.1 Limitations

Several limitations exist in this study. First, inherent biases such as self‐reporting and social desirability were unavoidable due to the subjective nature of a questionnaire‐based design, potentially affecting the accuracy of the data. To mitigate this, we emphasised anonymity and confidentiality during questionnaire distribution to encourage honest responses. Moreover, the study was conducted solely in a tertiary hospital in Jiaxing with a limited sample size. Therefore, the generalizability of the findings to hospitals at different levels or in other regions of the country may be limited. Another primary limitation of this study is the marginal fit observed in the RMSEA, which may be attributed to sample size and model complexity. Future studies should replicate this research in hospitals of varying levels across diverse geographical regions to provide a more comprehensive understanding, incorporating observational methods.

## Author Contributions

**Qianjuan Wang** and **Jiali Lu:** conceptualization, methodology, software. **Qianjuan Wang**, **Jiali Lu** and **Zhihong Zhu:** data curation, writing – original draught preparation. **Yuwei Wang**, **Liangfeng Zhu** and **Lan Shen:** writing – reviewing and editing.

## Funding

This study was funded by DCAF13‐mediated ubiquitination degradation of PAK1IP1 regulates the p53/p21 pathway to promote cellular senescence and liver cancer progression: Mechanism and its value as a clinical biomarker (LBY24H180006).

## Ethics Statement

The study was carried out after the protocol was approved by the hospital's Medical Ethics Committee (2023‐KY‐655). I confirm that all methods were performed in accordance with the relevant guidelines. All procedures were performed in accordance with the ethical standards laid down in the 1964 Declaration of Helsinki and its later amendments.

## Consent

Informed consent was obtained from all participants.

## Conflicts of Interest

The authors declare no conflicts of interest.

## Supporting information


**Data S1:** Supporting Information.

**Table S1:** Distribution of participants across Bloom's cut‐off categories for each KAP dimension.
**Table S2:** Distribution of knowledge responses.
**Table S3:** Distribution of attitude responses.
**Table S4:** Distribution of practise responses.
**Table S5:** Model fit indices for path analysis.

## Data Availability

All data generated or analysed during this study are included in this published article [and its Supporting Information files].
