# Correction to ‘Knowledge, Attitudes and Practices Among Nursing Staff in a Tertiary Hospital Regarding Subcutaneous Anticoagulant Administration in Jiaxing City, Southeast China’

**DOI:** 10.1002/nop2.70979

**Published:** 2026-11-01

Wang, Q. , J. Lu , Z. Zhu , Y. Wang , L. Zhu , and L. Shen . 2026. “Knowledge, Attitudes, and Practices Among Nursing Staff in a Tertiary Hospital Regarding Subcutaneous Anticoagulant Administration in Jiaxing City, Southeast China.” Nursing Open 00: 1–10. 10.1002/nop2.70687.PMC1336794242447276

In the original publication of this article, the funding information was incorrectly listed as:

‘This study was funded by DCAF13‐mediated ubiquitination degradation of PAK1IP1 regulates the p53/p21 pathway to promote cellular senescence and liver cancer progression: Mechanism and its value as a clinical biomarker (LBY24H180006)’.

The correct funding information should be:

Supported Discipline Project of Jiaxing, Surgery (Hepatobiliary and Pancreatic Subspecialty), Grant No. 2023‐ZC‐005.

Supported Discipline Project of Jiaxing, Nursing Science, Grant No. 2023‐ZC‐007.

We apologize for this error.

