# Supplementary material for: Knowledge, Attitudes and Practises Amongst Nursing Staff in a Tertiary Hospital Regarding Subcutaneous Anticoagulant Administration in Jiaxing City, Southeast China
Source: Nurs Open. 2026 Jul 14;13(7):e70687. doi: 10.1002/nop2.70687 (PMC13367942; doi:10.1002/nop2.70687)
Supplement: Supplementary file 1 — Data S1: Supporting Information. [file NOP2-13-e70687-s001.docx]

| Dear Nursing Colleague,  We are researchers from * Hospital, and we sincerely invite you to participate in our research project. The aim of this study is to understand the knowledge, attitudes, and practices of nursing personnel regarding subcutaneous injection of anticoagulant drugs. The information gathered will serve as a basis for developing scientific intervention strategies, potentially benefiting more individuals and improving their health conditions in the future. Your participation in this study is voluntary, and if you choose to join, please refer to the following instructions.  1. Please complete the questionnaire. There are no right or wrong answers; you only need to provide information based on your actual experiences. If you have any questions during the process, feel free to reach out to us. After completion, kindly submit the questionnaire promptly.  2. This research involves a simple survey and will not cause harm to your physical or psychological well-being. However, it may touch upon some privacy issues such as gender and age. Please rest assured that we will strictly maintain confidentiality and will not disclose your information.  3. As a participant, you are free to inquire about any information related to this study and its progress. If you decide to withdraw from the study, please inform us, and your data will not be included in the research results.  Finally, we sincerely appreciate your valuable time and support for our scientific research endeavors!  □ I am aware of and agree to allow the collected data to be used for scientific research.  Informed Consent Signature:  Date of Participation: year month day |
| --- |

| **Part I- basic information** | |
| --- | --- |
| **1.age：** | years |
| **2.gender** | a. male  b. female |
| **3.education：** | a. associate degree and below  b. bachelor’s degree  c. master’s degree and above |
| **4.years of work experience** | years |
| **5.your department：** | a. Obstetrics and Gynecology  b. Surgery  c. Orthopedics  d. Oncology  e. ICU  f. other |
| **6.professional title** | a. no title  b. junior  c. intermediate  d. senior |
| **7.** **The Grade of the Hospital Where You Work:** | a. Public tertiary hospital  b. Public secondary hospital  C. Public primary hospital  D. Private medical institution  E. other |
| **8.** **Is Your Workplace a Teaching Hospital?** | a. yes  b. no  c. not sure |
| **9.** **Have You Received Training on "Subcutaneous Injection of Anticoagulant Drugs"?** | a. yes  b. no |
| **10.** **Does Your Hospital Have Internal Guidelines for "Subcutaneous Injection of Anticoagulant Drugs"?** | a. yes  b. no  c. not sure |
| **11.** **In the Past Year, How Often Have You Performed Subcutaneous Injection of Anticoagulant Drugs?** | a. Less than once a week  b. 1-4 times per week  c. 5 times or more per week |

**Part II- knowledge**

**Please make your judgment based on your understanding of the question description by choosing "Correct," "Incorrect," or "Uncertain."**

| **1.** **Anticoagulant therapy is a crucial aspect of VTE prevention and treatment.** | **a. correct** | **b. incorrect** | **c. uncertain** |
| --- | --- | --- | --- |
| **2.** **Currently, anticoagulants available for subcutaneous injection mainly include low molecular weight heparins and fondaparinux.** | **a. correct** | **b. incorrect** | **c. uncertain** |
| **3.** **Mechanisms of heparin's antithrombotic action include:** |  |  |  |
| **Enhancing anticoagulant enzyme activity** | **a. correct** | **b. incorrect** | **c. uncertain** |
| **Inhibiting platelet function** | **a. correct** | **b. incorrect** | **c. uncertain** |
| **Enhancing protein C activity** | **a. correct** | **b. incorrect** | **c. uncertain** |
| **Promoting vasodilation** | **a. correct** | **b. incorrect** | **c. uncertain** |
| **4.** **The primary mechanism of fondaparinux's antithrombotic action is achieved by enhancing antithrombin III's neutralizing activity against factor Xa.** | **a. correct** | **b. incorrect** | **c. uncertain** |
| **5.** **The longer the injection needle for anticoagulants, the greater the risk when injecting into the muscle layer; therefore, shorter needles should be preferred whenever possible.** | **a. correct** | **b. incorrect** | **c. uncertain** |
| **6.** **Besides VTE prevention and treatment, indications for subcutaneous anticoagulant injection include:** |  |  |  |
| **Acute coronary syndrome** | **a. correct** | **b. incorrect** | **c. uncertain** |
| **Disseminated intravascular coagulation** | **a. correct** | **b. incorrect** | **c. uncertain** |
| **Ischemic stroke** | **a. correct** | **b. incorrect** | **c. uncertain** |
| **Hypertension** | **a. correct** | **b. incorrect** | **c. uncertain** |
| **Diabetic nephropathy** | **a. correct** | **b. incorrect** | **c. uncertain** |
| **7.** **Absolute contraindications for subcutaneous anticoagulant injection include:** |  |  |  |
| **Allergy to heparin or its derivatives** | **a. correct** | **b. incorrect** | **c. uncertain** |
| **Severe coagulation disorders** | **a. correct** | **b. incorrect** | **c. uncertain** |
| **Concurrent use of nonsteroidal anti-inflammatory drugs** | **a. correct** | **b. incorrect** | **c. uncertain** |
| **Active bleeding or organ injury with bleeding tendency** | **a. correct** | **b. incorrect** | **c. uncertain** |
| **Acute infective bacterial endocarditis** | **a. correct** | **b. incorrect** | **c. uncertain** |
| **Impaired liver or kidney function** | **a. correct** | **b. incorrect** | **c. uncertain** |
| **8.** **Potential risks of subcutaneous anticoagulant injection include:** |  |  |  |
| **Hematologic system abnormalities** | **a. correct** | **b. incorrect** | **c. uncertain** |
| **Immune system abnormalities** | **a. correct** | **b. incorrect** | **c. uncertain** |
| **Gastrointestinal system abnormalities** | **a. correct** | **b. incorrect** | **c. uncertain** |
| **Skin and subcutaneous tissue abnormalities** | **a. correct** | **b. incorrect** | **c. uncertain** |
| **Musculoskeletal system abnormalities** | **a. correct** | **b. incorrect** | **c. uncertain** |
| **9.** **Sites for subcutaneous anticoagulant injection primarily include the abdominal wall, upper 1/2 of the outer front side of both thighs, upper outer side of both buttocks, and the middle 1/2 of the outer side of the upper arms.** | **a. correct** | **b. incorrect** | **c. uncertain** |
| **10.** **Regularly rotating injection sites helps avoid bleeding and pain due to locally concentrated drug concentrations.** | **a. correct** | **b. incorrect** | **c. uncertain** |
| **11.** **Complications that may occur during subcutaneous anticoagulant injection include:** |  |  |  |
| **Subcutaneous bleeding** | **a. correct** | **b. incorrect** | **c. uncertain** |
| **Pain** | **a. correct** | **b. incorrect** | **c. uncertain** |
| **Leakage, seepage** | **a. correct** | **b. incorrect** | **c. uncertain** |
| **Allergic reactions** | **a. correct** | **b. incorrect** | **c. uncertain** |
| **Needle bending, breakage** | **a. correct** | **b. incorrect** | **c. uncertain** |
| **12.** **Are you familiar with the management strategies for the following complications?** |  |  |  |
| **Subcutaneous bleeding** | **a. familiar** | **b. partially familiar** | **c. unfamiliar** |
| **Pain** | **a. familiar** | **b. partially familiar** | **c. unfamiliar** |
| **Leakage, seepage** | **a. familiar** | **b. partially familiar** | **c. unfamiliar** |
| **Allergic reactions** | **a. familiar** | **b. partially familiar** | **c. unfamiliar** |
| **Needle bending, breakage** | **a. familiar** | **b. partially familiar** | **c. unfamiliar** |

**Part-III attitude**

**Please indicate your level of agreement with the statements by choosing from "Strongly Agree" to "Strongly Disagree."**

| **1.** **Subcutaneous anticoagulant injection is effective in preventing or treating blood clots.** | **a. strongly agree** | **b. agree** | **c. neutral** | **d. disagree** | **e. strongly disagree** |
| --- | --- | --- | --- | --- | --- |
| **2.** **Subcutaneous anticoagulant injection technique is an important component of nursing skills.** | **a. strongly agree** | **b. agree** | **c. neutral** | **d. disagree** | **e. strongly disagree** |
| **3.** **I wish to further study the relevant knowledge of subcutaneous anticoagulant injection.** | **a. strongly agree** | **b. agree** | **c. neutral** | **d. disagree** | **e. strongly disagree** |
| **4.** **I fully understand the requirements for subcutaneous anticoagulant injection and complication management.** | **a. strongly agree** | **b. agree** | **c. neutral** | **d. disagree** | **e. strongly disagree** |
| **5.** **Subcutaneous injection of anticoagulants should be performed according to standardized procedures.** | **a. strongly agree** | **b. agree** | **c. neutral** | **d. disagree** | **e. strongly disagree** |
| **6.** **Establishing relevant processes for subcutaneous anticoagulant injection is beneficial for better standardizing the quality of operations, ensuring homogeneity.** | **a. strongly agree** | **b. agree** | **c. neutral** | **d. disagree** | **e. strongly disagree** |
| **7.** **Establishing scoring standards for subcutaneous anticoagulant injection can better evaluate the operator's level.** | **a. strongly agree** | **b. agree** | **c. neutral** | **d. disagree** | **e. strongly disagree** |
| **8.** **Establishing scoring standards for subcutaneous anticoagulant injection can motivate nurses to improve their operational skills positively.** | **a. strongly agree** | **b. agree** | **c. neutral** | **d. disagree** | **e. strongly disagree** |
| **9.** **Mastering the correct subcutaneous anticoagulant injection skills is essential for improving patient experience.** | **a. strongly agree** | **b. agree** | **c. neutral** | **d. disagree** | **e. strongly disagree** |
| **10.** **Mastering the correct subcutaneous anticoagulant injection skills is essential for the effectiveness of anticoagulant therapy.** | **a. strongly agree** | **b. agree** | **c. neutral** | **d. disagree** | **e. strongly disagree** |

**Part IV- practice**

**Please select the most appropriate frequency based on how often you follow the specified procedures during the process of subcutaneous anticoagulant injections. Use the following reference for filling:**

**Always: Always perform the corresponding action every time. Often: Over 7-9 times out of every ten. Sometimes: 4-6 times out of every ten. Rarely: 1-3 times out of every ten. Never: Never perform the corresponding action every time.**

| **1.** **Introduce indications and contraindications of subcutaneous anticoagulant injection to patients.** | **a. always** | **b. often** | **c. sometimes** | **d. rarely** | **e. never** |
| --- | --- | --- | --- | --- | --- |
| **2.** **Inform patients (or their family members) about potential risks and precautions to alleviate anxiety and tension.** | **a. always** | **b. often** | **c. sometimes** | **d. rarely** | **e. never** |
| **3.** **Double-check patient identity, medication dosage, and name.** | **a. always** | **b. often** | **c. sometimes** | **d. rarely** | **e. never** |
| **4. Assess patient's physical condition (indications and contraindications), local conditions, psychological status, and cooperation level.** | **a. always** | **b. often** | **c. sometimes** | **d. rarely** | **e. never** |
| **5.** **Prepare for self-professional qualities, patient injection sites, injection environment, and injection materials.** | **a. always** | **b. often** | **c. sometimes** | **d. rarely** | **e. never** |
| **6** **Choose the injection position reasonably based on the patient's injection site.** | **a. always** | **b. often** | **c. sometimes** | **d. rarely** | **e. never** |
| **7.** **Use pre-filled anticoagulant syringes without the need for air expulsion.** | **a. always** | **b. often** | **c. sometimes** | **d. rarely** | **e. never** |
| **8.** **Use subcutaneous injection positioning cards and select injection sites in numerical order.** | **a. always** | **b. often** | **c. sometimes** | **d. rarely** | **e. never** |
| **9.** **Disinfect with the puncture point as the center, spiral disinfection, and allow natural drying.** | **a. always** | **b. often** | **c. sometimes** | **d. rarely** | **e. never** |
| **10.** **Pinch the skin to form a raised fold.** | **a. always** | **b. often** | **c. sometimes** | **d. rarely** | **e. never** |
| **11. Insert the needle quickly and vertically at the highest point of the fold, without drawing back blood.** | **a. always** | **b. often** | **c. sometimes** | **d. rarely** | **e. never** |
| **12.** **Slowly and evenly push the drug for 10 seconds, leave the needle in place for 10 seconds after the drug is injected, and quickly withdraw the needle without pressing.** | **a. always** | **b. often** | **c. sometimes** | **d. rarely** | **e. never** |
| **13.** **Properly place the patient and provide post-injection health education.** | **a. always** | **b. often** | **c. sometimes** | **d. rarely** | **e. never** |
| **14.** **Terminal processing, handwashing, recording, and signing.** | **a. always** | **b. often** | **c. sometimes** | **d. rarely** | **e. never** |
