# Supplementary material for: Knowledge, Attitudes and Practises Amongst Nursing Staff in a Tertiary Hospital Regarding Subcutaneous Anticoagulant Administration in Jiaxing City, Southeast China
Source: Nurs Open. 2026 Jul 14;13(7):e70687. doi: 10.1002/nop2.70687 (PMC13367942; doi:10.1002/nop2.70687)
Supplement: Supplementary file 2 — Table S1: Distribution of participants across Bloom's cut‐off categories for each KAP dimension. Table S2: Distribution of knowledge responses. Table S3: Distribution of attitude responses. Table S4: Distribution of practise responses. Table S5: Model fit indices for path analysis. [file NOP2-13-e70687-s002.docx]

Supplementary Table 1 Distribution of participants across Bloom’s cut-off categories for each KAP dimension

| Dimension | Category | Score ranges | n (%) |
| --- | --- | --- | --- |
| Knowledge | Poor (<60%) | <43.2 | 20 (3.7%) |
|  | Moderate (60–80%) | 43.2-57.6 | 222 (40.7%) |
|  | Good (>80%) | >57.6 | 303 (55.6%) |
| Attitude | Poor (<60%) | 10-29 | 6 (1.1%) |
|  | Moderate (60–80%) | 30-40 | 87 (16.0%) |
|  | Good (>80%) | >40 | 452 (82.9%) |
| Practice | Poor (<60%) | 14-41 | 8 (1.5%) |
|  | Moderate (60–80%) | 42-56 | 47 (8.6%) |
|  | Good (>80%) | >56 | 490 (89.9%) |

Supplementary table 2 Distribution of knowledge responses

| **Knowledge** | **N(%)** | | |
| --- | --- | --- | --- |
|  | **Correct** | **Incorrect** | **Uncertain** |
| **1.** **Anticoagulant therapy is a crucial aspect of VTE prevention and treatment.** | 540(99.08) | 4(0.73) | 1(0.18) |
| **2.** **Currently, anticoagulants available for subcutaneous injection mainly include low molecular weight heparins and fondaparinux.** | 500(91.74) | 22(4.04) | 23(4.22) |
| **3.** **Mechanisms of heparin's antithrombotic action include:** |  |  |  |
| **Enhancing anticoagulant enzyme activity** | 522(95.78) | 6(1.1) | 17(3.12) |
| **Inhibiting platelet function** | 456(83.67) | 49(8.99) | 40(7.34) |
| **Enhancing protein C activity** | 437(80.18) | 29(5.32) | 79(14.5) |
| **Promoting vasodilation*** | 363(66.61) | 97(17.8) | 85(15.6) |
| **4.** **The primary mechanism of fondaparinux's antithrombotic action is achieved by enhancing antithrombin III's neutralizing activity against factor Xa.** | 447(82.02) | 6(1.1) | 92(16.88) |
| **5.** **The longer the injection needle for anticoagulants, the greater the risk when injecting into the muscle layer; therefore, shorter needles should be preferred whenever possible.*** | 407(74.68) | 113(20.73) | 25(4.59) |
| **6.** **Besides VTE prevention and treatment, indications for subcutaneous anticoagulant injection include:** |  |  |  |
| **Acute coronary syndrome** | 482(88.44) | 25(4.59) | 38(6.97) |
| **Disseminated intravascular coagulation** | 443(81.28) | 79(14.5) | 23(4.22) |
| **Ischemic stroke** | 447(82.02) | 63(11.56) | 35(6.42) |
| **Hypertension*** | 276(50.64) | 183(33.58) | 86(15.78) |
| **Diabetic nephropathy** | 308(56.51) | 149(27.34) | 88(16.15) |
| **7.** **Absolute contraindications for subcutaneous anticoagulant injection include:** |  |  |  |
| **Allergy to heparin or its derivatives** | 528(96.88) | 6(1.1) | 11(2.02) |
| **Severe coagulation disorders** | 533(97.8) | 7(1.28) | 5(0.92) |
| **Concurrent use of nonsteroidal anti-inflammatory drugs*** | 370(67.89) | 100(18.35) | 75(13.76) |
| **Active bleeding or organ injury with bleeding tendency** | 520(95.41) | 18(3.3) | 7(1.28) |
| **Acute infective bacterial endocarditis** | 405(74.31) | 60(11.01) | 80(14.68) |
| **Impaired liver or kidney function*** | 387(71.01) | 92(16.88) | 66(12.11) |
| **8.** **Potential risks of subcutaneous anticoagulant injection include:** |  |  |  |
| **Hematologic system abnormalities** | 529(97.06) | 7(1.28) | 9(1.65) |
| **Immune system abnormalities** | 465(85.32) | 36(6.61) | 44(8.07) |
| **Gastrointestinal system abnormalities** | 492(90.28) | 25(4.59) | 28(5.14) |
| **Skin and subcutaneous tissue abnormalities** | 531(97.43) | 6(1.1) | 8(1.47) |
| **Musculoskeletal system abnormalities** | 429(78.72) | 59(10.83) | 57(10.46) |
| **9.** **Sites for subcutaneous anticoagulant injection primarily include the abdominal wall, upper 1/2 of the outer front side of both thighs, upper outer side of both buttocks, and the middle 1/2 of the outer side of the upper arms.** | 515(94.5) | 23(4.22) | 7(1.28) |
| **10.** **Regularly rotating injection sites helps avoid bleeding and pain due to locally concentrated drug concentrations.** | 539(98.9) | 4(0.73) | 2(0.37) |
| **11.** **Complications that may occur during subcutaneous anticoagulant injection include:** |  |  |  |
| **Subcutaneous bleeding** | 542(99.45) | 1(0.18) | 2(0.37) |
| **Pain** | 539(98.9) | 2(0.37) | 4(0.73) |
| **Leakage, seepage** | 531(97.43) | 9(1.65) | 5(0.92) |
| **Allergic reactions** | 537(98.53) | 4(0.73) | 4(0.73) |
| **Needle bending, breakage** | 533(97.8) | 10(1.83) | 2(0.37) |
|  | **a. familiar** | **b. partially familiar** | **c. unfamiliar** |
| **12.** **Are you familiar with the management strategies for the following complications?** |  |  |  |
| **Subcutaneous bleeding** | 466(85.5) | 75(13.76) | 4(0.73) |
| **Pain** | 463(84.95) | 79(14.5) | 3(0.55) |
| **Leakage, seepage** | 444(81.47) | 93(17.06) | 8(1.47) |
| **Allergic reactions** | 454(83.3) | 82(15.05) | 9(1.65) |
| **Needle bending, breakage** | 441(80.92) | 96(17.61) | 8(1.47) |

* indicated the correct response to the item was “incorrect”.

Supplementary table 3 Distribution of attitude responses

| **Attitude** | **Strongly agree** | **Agree** | **Neutral** | **Disagree** | **Strongly disagree** |
| --- | --- | --- | --- | --- | --- |
| **1.** **Subcutaneous anticoagulant injection is effective in preventing or treating blood clots.** | 420(77.06) | 117(21.47) | 7(1.28) |  | 1(0.18) |
| **2.** **Subcutaneous anticoagulant injection technique is an important component of nursing skills.** | 421(77.25) | 111(20.37) | 12(2.2) |  | 1(0.18) |
| **3.** **I wish to** **further study the relevant knowledge of subcutaneous anticoagulant injection.** | 404(74.13) | 130(23.85) | 10(1.83) |  | 1(0.18) |
| **4.** **I fully understand the requirements for subcutaneous anticoagulant injection and complication management.** | 332(60.92) | 161(29.54) | 48(8.81) | 4(0.73) |  |
| **5.** **Subcutaneous anticoagulant administration should be performed according to standardized procedures.** | 438(80.37) | 96(17.61) | 11(2.02) |  |  |
| **6.** **Establishing relevant processes for subcutaneous anticoagulant injection is beneficial for better standardizing the quality of operations, ensuring homogeneity.** | 436(80) | 98(17.98) | 11(2.02) |  |  |
| **7.** **Establishing scoring standards for subcutaneous anticoagulant injection can better evaluate the operator's level.** | 431(79.08) | 103(18.9) | 11(2.02) |  |  |
| **8.** **Establishing scoring standards for subcutaneous anticoagulant injection can motivate nurses to improve their operational skills positively.** | 419(76.88) | 108(19.82) | 16(2.94) | 1(0.18) | 1(0.18) |
| **9.** **Mastering the correct subcutaneous anticoagulant injection skills is essential for improving patient experience.** | 437(80.18) | 98(17.98) | 10(1.83) |  |  |
| **10.** **Mastering the correct subcutaneous anticoagulant injection skills is essential for the effectiveness of anticoagulant therapy.** | 435(79.82) | 99(18.17) | 11(2.02) |  |  |

Supplementary table 4 Distribution of practice responses

| **Practice** | **Always** | **Often** | **Sometimes** | **Rarely** | **Never** |
| --- | --- | --- | --- | --- | --- |
| **1.** **Introduce indications and contraindications of subcutaneous anticoagulant injection to patients.** | 391(71.74) | 115(21.1) | 33(6.06) | 5(0.92) | 1(0.18) |
| **2.** **Inform patients (or their family members) about potential risks and precautions to alleviate anxiety and tension.** | 401(73.58) | 111(20.37) | 28(5.14) | 4(0.73) | 1(0.18) |
| **3.** **Double-check patient identity, medication dosage, and name.** | 482(88.44) | 50(9.17) | 11(2.02) | 1(0.18) | 1(0.18) |
| **4. Assess patient's** **physical condition (indications and contraindications), local conditions, psychological status, and cooperation level.** | 433(79.45) | 90(16.51) | 19(3.49) | 2(0.37) | 1(0.18) |
| **5.** **Prepare for self-professional qualities, patient injection sites, injection environment, and injection materials.** | 459(84.22) | 74(13.58) | 10(1.83) | 1(0.18) | 1(0.18) |
| **6** **Choose the injection position reasonably based on the patient's injection site.** | 468(85.87) | 67(12.29) | 8(1.47) | 1(0.18) | 1(0.18) |
| **7.** **Use pre-filled anticoagulant syringes without the need for air expulsion.** | 475(87.16) | 55(10.09) | 10(1.83) | 1(0.18) | 4(0.73) |
| **8.** **Use subcutaneous injection positioning cards and select injection sites in numerical order.** | 426(78.17) | 81(14.86) | 27(4.95) | 5(0.92) | 6(1.1) |
| **9.** **Disinfect with the puncture point as the center, spiral disinfection, and allow natural drying.** | 482(88.44) | 51(9.36) | 10(1.83) | 1(0.18) | 1(0.18) |
| **10.** **Pinch the skin to form a raised fold.** | 479(87.89) | 55(10.09) | 7(1.28) | 2(0.37) | 2(0.37) |
| **11. Insert the needle quickly and vertically at the highest point of the fold, without drawing back blood.** | 473(86.79) | 50(9.17) | 9(1.65) | 1(0.18) | 12(2.2) |
| **12.** **Slowly and evenly push the drug for 10 seconds, leave the needle in place for 10 seconds after the drug is injected, and quickly withdraw the needle without pressing.** | 474(86.97) | 51(9.36) | 9(1.65) | 4(0.73) | 7(1.28) |
| **13.** **Properly place the patient and provide post-injection health education.** | 472(86.61) | 58(10.64) | 12(2.2) | 2(0.37) | 1(0.18) |
| **14.** **Terminal processing, handwashing, recording, and signing.** | 482(88.44) | 51(9.36) | 10(1.83) | 1(0.18) | 1(0.18) |

Supplementary Table 5. Model fit indices for path analysis

| Fit Index | Reference Criterion | Result | Interpretation |
| --- | --- | --- | --- |
| RMSEA | < 0.08 (Good fit) | 0.126 | Marginal fit |
| SRMR | < 0.08 (Good fit) | 0.068 | Good fit |
| TLI | > 0.80 (Good fit) | 0.826 | Good fit |
| CFI | > 0.80 (Good fit) | 0.923 | Good fit |
